# Modelling DTPA therapy following Am contamination in rats

**DOI:** 10.1007/s00411-023-01046-z

**Published:** 2023-10-13

**Authors:** Manuel Kastl, Olivier Grémy, Stephanie Lamart, Augusto Giussani, Wei Bo Li, Christoph Hoeschen

**Affiliations:** 1 Institute of Radiation Medicine, Helmholtz Center Munich, German Research Center for Environmental Health, Neuherberg, Germany; 2 Laboratoire de Radio Toxicologie, CEA, Université de Paris-Saclay, Arpajon, France; 3 Laboratoire d’Evaluation de la Dose Interne, Institut de Radioprotection et de Sûreté Nucléaire (IRSN), PSE-SANTE/SDOS/LEDI, Fontenay-aux-Roses, France; 4 Division of Medical and Occupational Radiation Protection, Federal Office for Radiation Protection, Oberschleißheim, Germany; 5 Institut für Medizintechnik, Otto-Von-Guericke University Magdeburg, Magdeburg, Germany

**Keywords:** Chelation, DTPA, Americium, Biokinetic model, Decorporation

## Abstract

A major challenge in modelling the decorporation of actinides (An), such as americium (Am), with DTPA (diethylenetriaminepentaacetic acid) is the fact that standard biokinetic models become inadequate for assessing radionuclide intake and estimating the resulting dose, as DTPA perturbs the regular biokinetics of the radionuclide. At present, most attempts existing in the literature are empirical and developed mainly for the interpretation of one or a limited number of specific incorporation cases. Recently, several approaches have been presented with the aim of developing a generic model, one of which reported the unperturbed biokinetics of plutonium (Pu), the chelation process and the behaviour of the chelated compound An–DTPA with a single model structure. The aim of the approach described in this present work is the development of a generic model that is able to describe the biokinetics of Am, DTPA and the chelate Am–DTPA simultaneously. Since accidental intakes in humans present many unknowns and large uncertainties, data from controlled studies in animals were used. In these studies, different amounts of DTPA were administered at different times after contamination with known quantities of Am. To account for the enhancement of faecal excretion and reduction in liver retention, DTPA is assumed to chelate Am not only in extracellular fluids, but also in hepatocytes. A good agreement was found between the predictions of the proposed model and the experimental results for urinary and faecal excretion and accumulation and retention in the liver. However, the decorporation from the skeletal compartment could not be reproduced satisfactorily under these simple assumptions.

**Supplementary Information:**

The online version contains supplementary material available at 10.1007/s00411-023-01046-z.

## Introduction

The incorporation of actinides (An), e.g. plutonium (Pu) or americium (Am), can cause severe health damage due to the continuous alpha irradiation of organs and tissues such as the lungs, liver and skeleton, where they can be retained for decades (ICRP 2019). Treatment with the chelating agent DTPA (diethylenetriaminepentaacetic acid) as a salt of calcium (Ca) or zinc (Zn) is the commonly used therapy to remove Pu/Am from the body (Ménétrier et al. 2005; Grappin and Bérard 2008; Grappin et al. 2006, 2007a, b, 2009). Indeed, the injection or inhalation of Ca/Zn–DTPA enhances the excretion of the incorporated actinide by forming stable An–DTPA chelates, thus minimizing the amount of An retained in the body and the resulting committed effective dose.

The basic principle of decorporation therapy with chelating agents is described in (Kety 1942) and has been applied since the 1950s (Catsch 1968; Volf 1978). Ca–DTPA has been approved for the treatment of Pu/Am incorporation in many countries, e.g. in the USA (FDA 2015), France (Grappin and Bérard 2008) and Germany (approved in 2005, marketing authorization number 6813281.00.00^1^). Numerous animal studies and human studies have provided evidence about the efficacy of DTPA treatment (Volf 1978; Catsch 1968; Roedler et al. 1989; Carbaugh et al. 1989; Grémy et al. 2016, 2021; Jech et al. 1972; Fisher 2000; Bhattacharyya et al.^1992^; Bertelli et al. 2010, 2018; Breustedt et al. 2019; Cohen et al. 1974; Davesne et al. 2016; Hengé-Napoli et al. 2000; Grappin et al. 2006; Gorden et al. 2003; Durbin et al. 2006; Stradling et al. 2000; Fritsch et al. 2007; James et al. 2007; Jolly et al. 1972; Poudel et al. 2017; Schadilov 2010; Schadilov et al. 2005; Schofield and Lynn 1973; Norwood 1960; Ohlenschläger et al. 1978; Lamart et al. 2021). Side effects and complications associated with DTPA treatments are not uncommon (Taylor et al. 2007; Glover et al. 2022).

Usually, the dose after the incorporation of radionuclides is estimated by interpreting direct measurements of the activity in the body or by measurements of the excreted activity using compartment models. These models provide a mathematical method to predict the distribution and excretion patterns of the radionuclides in the body (ICRP 2015).

In the case of chelation therapy, the complexation between the chelating agent and actinide perturbs the characteristic biokinetic behaviour of the actinide because the resulting An–DTPA chelate is very readily and rapidly excreted. Consequently, the existing reference biokinetic models, such as those published by the International Commission on Radiation Protection (ICRP 2019), become inadequate for interpreting bioassay measurements after the administration of DTPA, estimating the incorporated activity and assessing the success of the therapy in terms of averted dose.

However, estimates of the incorporated activities and of the resulting doses are necessary for planning individually adapted therapies. This can be achieved with a generic model able to predict the effects of DTPA on the biokinetics of actinides depending on, e.g. the type, amount and schedule of administration. The optimization of the treatment protocols involves balancing the gains in terms of the averted dose and the resulting health benefits for the contaminated persons with the efforts needed for performing the therapy, including financial costs, time burden, as well as discomfort and side effects for the treated individuals. Numerous modelling approaches are reported in the literature; however, most of them are case specific and empirical and do not provide an explicit description of the chelation processes (Jech et al. 1972; Jolly et al. 1972; Poudel et al. 2017; Hall et al. 1978; LaBone 1994, 2002; Bailey et al. 2003; Fritsch et al. 2007, 2009; James et al. 2007; Breustedt et al. 2019; Serandour and Fritsch 2008; Konzen et al. 2016). They are not suitable for general predictions of therapeutic success in connection with DTPA, since these approaches are optimized for specific experimental results. Recent works based on human data pursued the aim of developing a generic model: Konzen and Brey (2015) provide a Pu–DTPA biokinetic model for the estimation of Pu intake, which can be used to evaluate several chelation strategies and, hence, derive some recommendations for effective treatment. Dumit et al. (2019a, b) presented an attempt to provide a comprehensive description of the unperturbed biokinetics of Pu, the chelation process and the behaviour of the chelated compound Pu–DTPA with a single model structure using the so-called CONRAD approach, first described in Breustedt et al. (2009). This approach consists of modelling the biokinetic behaviour of the actinide, of the intravenously injected chelating agent and of the in vivo formed chelate as separate structures which are then coupled together by a suitable mathematical description of the chelation mechanism as a second-order process.

A major issue in modelling decorporation therapies with chelating agents is the difficulty in deriving information about the chelation site from the available data. To date, no mechanisms are known that would allow DTPA to cross the cell membranes and enter the cells. A frequently used assumption is therefore that DTPA is distributed only in the extracellular fluids (ECF). This approach is supported by the particularly high efficiency of DTPA therapies observed when DTPA was administered shortly after the accidental incorporation, while the actinide was still present in the plasma and ECF, and by the fact that more than 99% of DTPA was excreted from the body within the first day after injection (Breustedt et al. 2009; Stevens et al. 1978; Stather et al. 1983; Durbin et al. 1997).

On the other hand, several studies have indicated liver and skeletal decorporation of Pu/Am in animals (Bhattacharyya et al. 1978a, b; Bhattacharyya and Peterson 1979; Cohen et al. 1974; Fritsch et al. 2010; Grémy et al. 2016) and humans (Roedler et al. 1989; Fritsch et al. 2007; James et al. 2007; Grémy et al. 2021; Dumit et al. 2019a, b) and reported an effectiveness of late treatment (Kety 1942; Roedler et al. 1989; Volf et al. 1999; James et al. 2007; Grémy et al. 2021), when the actinide is assumed not to be in the ECF any longer. Other animal studies documenting an increased biliary/faecal excretion of Pu/Am postulate that DTPA is able to penetrate the liver cell boundaries and suggest intracellular chelation (Schubert et al. 1961; Grémy et al. 2016; Markley et al. 1964; Ballou and Hess 1972; Bhattacharyya et al. 1978a, b; Bhattacharyya and Peterson 1979). Here, it should be noted that the amount of DTPA administered in animal studies generally by far (several orders of magnitude) exceeds the amount of actinide in terms of moles. Recent works from Dumit et al. have used a comprehensive amount of human data to develop and validate chelation models (Dumit et al. 2019a, b, 2020a, b, 2023). These publications show evidence of intracellular chelation in both skeleton and liver. The present work, however, uses animal data and it is not straightforward to reproduce and observe the same results obtained when using human data. Based on this evidence, the possibility that a very small fraction of DTPA is able to cross cell membranes and chelate material deposited there has been intensively discussed (Grémy et al. 2016, 2021; Fritsch et al. 2010; Grémy and Miccoli 2019).

Alternatively, the increased faecal excretion might be explained by a mobilization of nonchelated actinides in the liver after DTPA administration (Hall et al. 1978), leaving the site of chelation as a fundamentally open question, which cannot be solved only based on data interpretation. However, the development of adequate biokinetic models can contribute to solving this question.

Human data are usually derived from accidental incorporation cases, for which the type and chemical form of the incorporated material are in general known only very roughly, if ever, and sometimes even the exact time of incorporation is unknown. More critically, the transfer of actinides from the primary site of incorporation (wound site for injuries/lung for inhalation) to the systemic circulation (input function into blood compartment), which is crucial information for the proper modelling of the chelation processes, is not known. Trying to derive this information using generic models with reference parameter values, i.e. not specific for the individual case under study, will merely introduce a perturbing bias in the analysis. The many unknowns and uncertainty sources associated with human incorporation cases make it difficult if not impossible to identify the chelation sites and estimate the values of the rate constants.

An alternative to using human data is the use of experimental data from controlled animal studies, for which all boundary conditions are known, thus leaving the definition of the sites of chelation and chelation rate constants as the only real unknown variables of the system. In the present work, models for describing the mechanism of the DTPA decorporation of Am in contaminated rats have been developed based mainly on results from rat studies conducted in the Laboratory of Radio Toxicology (LRT) of the French Commission of Atomic Energy and Alternative Energies (Commissariat à l’Energie Atomique et aux Energies Alternatives, CEA, France). Part of these data related to rats contaminated with Am–citrate with or without treatment through a single intravenous injection of different quantities of DTPA at different times before or after contamination (prophylactic and delayed treatments) was published in Grémy et al. (2016). The present work focuses exclusively on data from delayed treatment studies, in which DTPA was administered at Day 1 after Am contamination or later, i.e. at a time when Am content in blood is negligible with respect to the early post-contamination phase. This set of data thus enabled us to focus our attention on the chelation mechanisms for Am not in blood, i.e. on the long-term effects of DTPA decorporation studies.

Adopting the CONRAD approach (Breustedt et al. 2009), simplified systemic models for the kinetics of Am–citrate and DTPA in rats were developed. In addition to the assumption that chelation can occur only in the ECF, hepatocytes were also considered a possible chelation site to account for the reduction in liver burden and the enhancement of faecal excretion. The future aim is to transfer the gained knowledge to the human model and apply this knowledge to the existing human incorporation cases.

## Materials and methods

### Data

The models were fit to literature data and archive data (unpublished) provided by CEA/LRT in the framework of the EURADOS collaboration. An outline of the type of data and of the relative studies is given below. The whole set of data is made available as supplementary material.

The Am studies at CEA/LRT were all performed under the same experimental conditions, administering 9.42 kBq Am–citrate to each rat. The precise description of the experimental setup of these studies and additional details on the used solutions, animal housing setup, contamination procedure, chelation treatment and collection and measurement of urine, faeces and tissue samples are the same as those documented in Grémy et al. (2016).﻿

#### Americium citrate (controls)

- Uptake in and clearance from blood (Table A1): data from the CEA/LRT studies and Turner and Taylor (1968). Data are expressed as the percentage of the injected activity (% IA; mean ± SD).
- Uptake in liver and the skeleton: data from the studies conducted at CEA/LRT. Organ retention on Day 14 was 7.06 ± 0.99% for the liver and 46 ± 3% for the skeleton. Data are expressed as the percentage of the injected activity (% IA; mean ± SD; *n* = 4).
- Am excretion in urine and faeces (Tables A2 and A3): data from the studies conducted at CEA/LRT; data are given as 24 h cumulative excretion, expressed as the percentage of the injected activity (% IA; mean ± SD).

#### DTPA

Data on the kinetics of DTPA were obtained from unpublished studies conducted at CEA/LRT. Pu–DTPA chelates were prepared in vitro at a concentration equivalent to a DTPA dose of 30 µmol·kg^−1^ and were administered intravenously in male Sprague–Dawley rats (12.2 kBq in 200 µl).

- Uptake in and clearance from blood: data from the CEA/LRT studies (Table A4). Data are expressed as the percentage of the injected activity (% IA). A standard deviation of 30% was assumed for the Pu–DTPA data. This value was found to be a typical standard deviation for plasma clearance in other similar experiments conducted at CEA/LRT.
- DTPA elimination in urine: data from the CEA/LRT studies (Table A5). Data are presented as cumulative excretion expressed as the percentage of the injected activity (% IA). The standard deviation was set equal to the measurement error, or to 3% of the measured value (whichever the highest).
- DTPA elimination in faeces: although the experimental evidence on DTPA in humans and animals shows that more than 99% is excreted very rapidly in urine, tiny amounts of injected [^14^C]DTPA were still detected in the bile of rats (0.12% at 24 h) (Bhattacharyya and Peterson 1979), which was associated with faecal excretion.

Additional data on the kinetics of DTPA complexes (^99m^Tc–DTPA and ^153^Gd–(DTPA)^2−^) in blood and urine, presented by Wedeking et al. (1990), were used to check the model predictions. A special feature of this dataset is that the blood clearance was studied in detail in the first few minutes after administration. The uncertainty of the blood data corresponds to the 95% confidence interval for each data point, as given in the original reference. The urinary excretion data (Table 2 in Wedeking et al. (1990)) are given as the percentage of injected activity (% IA; mean ± SD; *n* = 6).

#### DTPA decorporation studies

Data from three decorporation studies conducted at CEA/LRT were used. In these experiments, rats were administered 300 µmol DTPA kg^−1^ body weight on Day 1 (Experiment A), 3 (Experiment B) and 7 (Experiment C) after contamination with 9.42 kBq Am–citrate.

- Uptake in liver and the skeleton (Table A6: data from the studies conducted at CEA/LRT; data for organ retention on Day 14 are expressed as the mean percentage of the injected activity (% IA; mean ± SD; *n* = 4).
- Am excretion in urine and faeces: (Tables A7 and A8: data from the studies conducted at CEA/LRT; Data are given as 24 h cumulative excretion, expressed as the percentage of the injected activity (% IA; mean ± SD; *n* = 4).

### Chelation modelling and model assumptions

In biokinetic models used in radiation protection, human organs and tissues are generally simplified as separate compartments, and the translocations of materials between the organs and tissues are described by transfer rates. These biokinetic models are used to describe the time-dependent activity of a given incorporated radionuclide in the biological compartments.

After chelation therapy, the model should describe simultaneously the biokinetics not of one, but of three forms: the incorporated Am, the DTPA and the Am–DTPA chelate. The CONRAD approach relies on the simultaneous use of three separate model structures to describe the biokinetic behaviour of the three available forms (Breustedt et al. 2009). As the kinetics of the Am–DTPA chelate are assumed to be the same as the kinetics of DTPA, the model structures of these two forms are identical. The three structures are combined together assuming that the in vivo﻿ chelation of Am by DTPA is proportional to the concentrations of both DTPA and actinide, mathematically described as a second-order kinetics process.

Assuming chelation between Am in a given compartment $j$ of the Am model structure and DTPA in a given compartment $i$ of the DTPA model structure, the formation of Am–DTPA chelates in compartment $i$ can be written as

$$\frac{\mathrm{d}{z}_{i}}{\mathrm{d}t}=-\sum_{h=1}^{m}{k}_{hi}{z}_{i}+\sum_{h=1}^{m}{k}_{ih}{z}_{h}+\sum_{j=1}^{n}k{R}_{ij}\cdot f({x}_{j},{y}_{i})= \sum_{h=1}^{m}\left({k}_{ih}{z}_{h}-{k}_{hi}{z}_{i}\right)+\sum_{j=1}^{n}k{R}_{ij}\cdot f\left({x}_{j},{y}_{i}\right),$$

with

$$f\left({x}_{j},{y}_{i}\right)={x}_{j}\cdot {y}_{i}$$

and

- ${x}_{j}$ is the time-dependent content of the *j*th compartment in the Am model [in moles];
- ${y}_{i}$ is the time-dependent content of the *i*th compartment in the Am model [in moles];
- ${z}_{h}$ is the time-dependent content of the *h*th compartment in the Am model [in moles].

The sum index $h$ runs from 1 to $m$ (number of compartments in the systemic model of DTPA, the same as in the model of Am–DTPA), ${k}_{hi}$ is the transfer coefficient describing passage from compartment $i$ to compartment $h$ of the DTPA model (same as for Am–DTPA) and the last sum is over all compartments ${x}_{j}$ of the Am model where chelation with DTPA in compartment $i$ can occur.

Consequently, the equation for DTPA (${y}_{i}$) in compartment $i$ is given by

$$\frac{{\mathrm{d}y}_{i}}{\mathrm{d}t}=-\sum_{h=1}^{m}{k}_{hi}{y}_{i}+\sum_{h=1}^{m}{k}_{ih}{y}_{h}- \sum_{j=1}^{n}k{R}_{ij}\cdot f\left({x}_{j},{y}_{i}\right)= \sum_{h=1}^{m}\left({{k}_{ih}{y}_{h}-k}_{hi}{y}_{i}\right)-\sum_{j=1}^{n}k{R}_{ij}\cdot f\left({x}_{j},{y}_{i}\right),$$

and the equation for Am in compartment $j$ (${x}_{j}$) is given by

$$\frac{\mathrm{d}{x}_{j}}{\mathrm{d}t}=-\sum_{l=1}^{n}{k}_{lj}^{Am}{x}_{j}+\sum_{l=1}^{n}{k}_{jl}^{Am}{x}_{l}-\sum_{i=1}^{m}k{R}_{ij}\cdot f\left({x}_{j},{y}_{i}\right) = \sum_{l=1}^{n}\left({k}_{jl}^{Am}{x}_{l}-{k}_{lj}^{Am}{x}_{j}\right)-\sum_{i=1}^{m}k{R}_{ij}\cdot f\left({x}_{j},{y}_{i}\right),$$

where the sum index $l$ runs from 1 to $n$ (the number of compartments in the systemic model of Am), ${k}_{hj}^{Am}$ is the transfer coefficient describing passage from compartment $j$ to compartment $h$ of the Am model and the last sum is over all compartments ${y}_{i}$ of the DTPA model, where the chelation of Am with DTPA in compartment $i$ can occur.

As one DTPA molecule can chelate one Am ion, the Am and DTPA entities in a given chelation site both disappear with the same rate ${kR}_{ij}\cdot {x}_{j}\cdot {y}_{i}$, proportional to the rate constant ${kR}_{ij}$ and to the molar contents of Am and DTPA in the sites where chelation occurs. The Am–DTPA chelate appears in the corresponding site of DTPA and with the same chelation rate.

### Model implementation and fitting

The model structures and the underlying equations were implemented using SAAM II software (©The Epsilon Group, Charlottesville, Virginia, USA). SAAM II is a software package that is applied to build models, run simulations and analyse results. The values of the adjustable parameters were estimated using the fitting tools and algorithms available in the SAAM II software package. The objective function to be minimized is defined in the SAAM software as the extended least-squares maximum likelihood function. It depends among others on the residuals, i.e. the differences between the model predictions and the experimental data, and on the variance associated with them (Barrett et al. 1998). The variance model was chosen, to reflect different levels of reliability of the different types of data available.

Other indicators of the goodness of fit used in addition to the convergence criterion were:

- The coefficient of variation of the parameters, as calculated by the software, that corresponds to 1 SD and is expressed as a fraction of the parameter estimate.
- The correlation coefficients used to identify any correlations between the parameters and therefore the possibility of simplifying the structure of the model, to pursue the principle of model parsimony.
- The Akaike information criterion [AIC] and the Bayesian information criterion [BIC], which were used to compare competing structures (again in compliance with the parsimony principle).

A fit was therefore considered successful based on the combination of these different indicators.

First, the model structures had to be defined, and all the model parameters were considered to be “adjustable”. They were estimated separately for the Am and DTPA models using the respective data (see “Materials and methods”, Tables in the supplementary material). Thereafter, the calculated transfer coefficients were kept fixed, while the only unknown parameters, the chelation rate constants kR_ij_, were fitted simultaneously to the overall dataset of all decorporation experiments (A, B and C). For the model analysis, the cumulative activity over the entire measurement period was calculated by the summation of the daily excreted activities.

The identification of the chelation sites and the chelation rate constants are thus the only unknown variables of the system.

## Results

### DTPA biokinetic model for rats

**Fig. 1:**
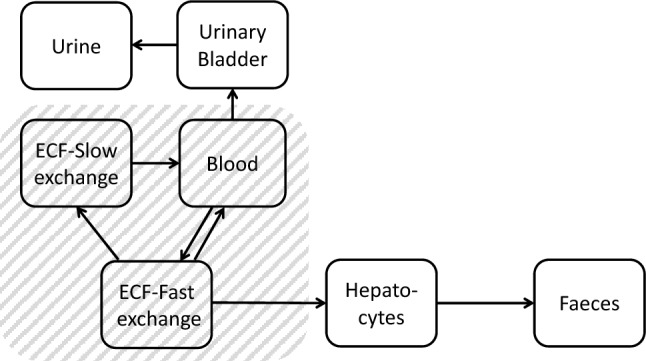
Compartmental model of DTPA kinetics in rats. ECF stands for extracellular fluids. The shaded area corresponds to the circulating DTPA

The model structure for DTPA kinetics in rats is based on the one previously developed by Breustedt et al. (2009), starting from human data presented by Stather et al. (1983). Based on the information that 0.12% of the injected [^14^C]DTPA was excreted into bile by 24 h after DTPA administration, the ability of DTPA to enter hepatocytes to a small extent prior to its biliary/faecal elimination (Stevens et al. 1978; Bhattacharyya et al. 1978a; Bhattacharyya and Peterson 1979; Ballou and Hess 1972) can be assumed. This is in accordance with the fact that delayed DTPA treatments enhanced biliary/faecal clearance of Pu/Am in rats (Bhattacharyya et al. 1978a; Bhattacharyya and Peterson 1979; Ballou and Hess 1972), dogs (Stevens et al. 1978; Grémy et al. 2016), pigs (Smith et al. 1961) and humans (Norwood 1960; Roedler et al. 1989; James et al. 2007; Grémy et al. 2021, 2022). To take this into account, the structure was modified by adding a compartment to represent hepatocytes and a new path to faecal loss to reproduce the excretion of DTPA chelates in faeces (Fig. 1).

The parameter values of the modified model were estimated based on the CEA/LRT data on Pu–DTPA kinetics in rats (Sect. “DTPA”) and are shown in Table 1. In line with the approach pursued by the CONRAD project and with the available data, it is assumed that the biokinetic behaviour of DTPA complexes is independent of the ligand due to the comparatively large dimension of the DTPA molecule. Furthermore, no dependence is assumed on the ionic charge.

**Table 1:** Parameters of the model in Fig. 1

Pathway	Transfer rate value (d^−1^)
Urinary Bladder → Urine	(1.5 ± 0.4) · 10^2^
Blood → Urinary Bladder	(2.85 ± 0.06) · 10^2^
ECF-Fast exchange → Blood	(3.1 ± 0.2) · 10^2^
ECF-Slow exchange → Blood	(8.0 ± 0.5) · 10^–1^
Blood → ECF-Fast exchange	(1.15 ± 0.05) · 10^3^
ECF-Fast exchange → ECF-Slow exchange	(1.20 ± 0.08) · 10^1^
ECF-Fast exchange → Hepatocytes	(9.7 ± 1.0) · 10^–2^
Hepatocytes → Faeces	(9.48 ± 0.22) · 10^–1^

The identification of the two compartments representing the ECF as *interstitial fluids* and *lymph*, made in Breustedt et al. (2009), merely reflects a physiological assumption and cannot be substantiated by experimental data. For this reason, these compartments are indicated here as "ECF-Fast exchange” and “ECF-Slow exchange" in Fig. 1.

**Fig. 2:**
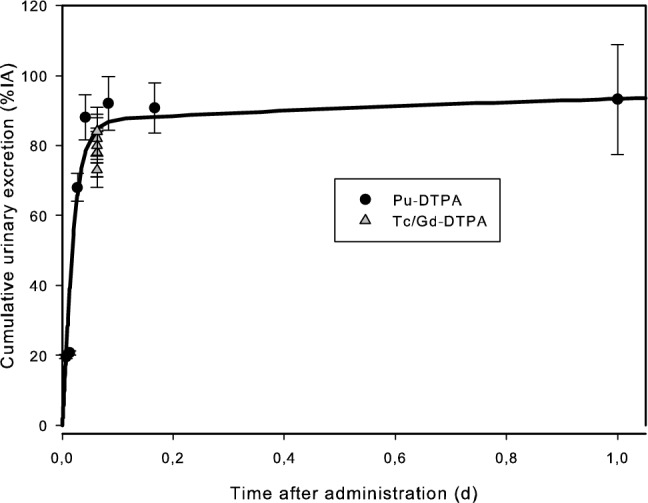
Cumulative urinary excretion of DTPA in rats. Solid line: model prediction; black dots: Pu–DTPA, provided by CEA/LRT; grey triangles: Tc/Gd–DTPA, from Wedeking et al. (1990)

**Fig. 3:**
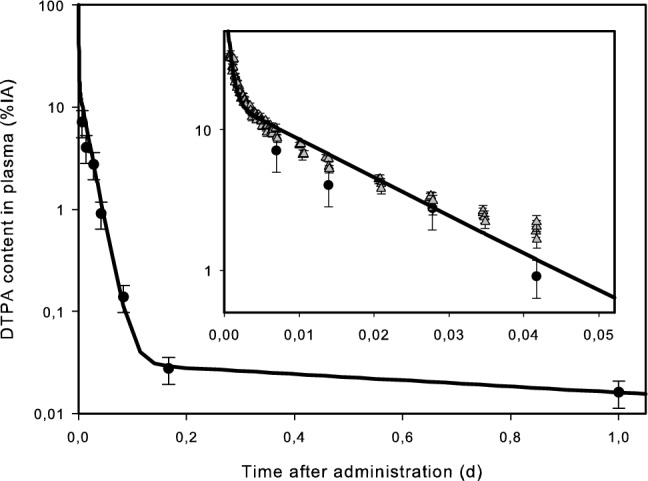
Clearance of DTPA in blood of rats. Solid line: model prediction; black dots: Pu–DTPA, provided by CEA/LRT; grey triangles: Tc/Gd–DTPA, from Wedeking et al. (1990)

Figures 2 and 3 show the experimental data of the CEA/LRT studies and the corresponding DTPA model predictions for urine and plasma, respectively. For comparison, the data from Wedeking et al. (1990), not used for the model fitting, are also shown.

### Americium rat model

**Fig. 4:**
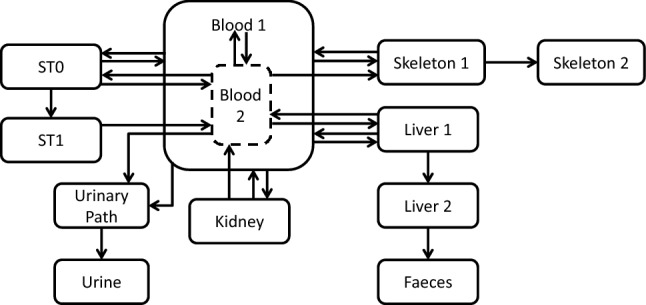
Compartmental model of Am kinetics in rats

The model structure for Am biokinetics in rats, shown in Fig. 4, was developed based on the knowledge that Am preferably accumulates in the liver and skeleton. Am in blood plasma is divided into two compartments (Blood1 and Blood2) to consider short and long retention of Am in plasma. Both plasma compartments exchange material with identified organs and tissues: the skeleton, liver and kidney. Other organs and tissues to which Am can be transferred were pooled into two generic soft tissue compartments (ST0 and ST1). Faecal excretion is channelled directly from the liver (Liver 2).

The overall model structure was developed using the complete available dataset of the control rats (Sect. “Americium citrate (controls)”). Table 2 shows the parameter values corresponding to the best fit to the available data. These were then kept fixed in the next step of the analysis of the chelation study data.

**Table 2:** Parameters of the model in Fig. 4

Pathway	Transfer rate value (d^−1^)
Blood1—> Liver1	(7.1 ± 0.2) · 10^4^
Blood2—> Blood1	(5.4 ± 0.6)
Blood1—> Blood2	(5.4 ± 0.3) · 10^4^
Blood2—> Liver1	(6.7 ± 0.5) · 10^–1^
Liver1—> Blood1	(7.0 ± 0.9) · 10^–2^
Liver1—> Liver2	(1.35 ± 0.05) · 10^–1^
Liver2—> Faeces	(5.1 ± 0.2) · 10^–1^
Blood1—> ST0	(2.5 ± 0.2) · 10^–2^
Blood2—> ST0	(6.0 ± 0.2) · 10^1^
ST0—> Blood1	(3.6 ± 5.3) · 10^–3^
ST0—> Blood2	(18.1 ± 1.8)
ST0—> ST1	(9.4 ± 1.0)
ST1—> Blood2	(2.81 ± 0.02)
Blood1—> Skeleton1	(1.7 ± 1.4) · 10^–1^
Blood2—> Skeleton1	(4.26 ± 0.02) · 10^1^
Skeleton1—> Blood1	(2.18 ± 0.11) · 10^–1^
Skeleton1—> Skeleton2	(1.1 ± 0.2)
Kidney—> Blood1	(2.93 ± 0.17) · 10^–2^
Kidney—> Blood2	(6.3 ± 0.4) · 10^–2^
Blood1—> Kidney	(3.6 ± 0.4) · 10^4^
Blood1—> Urinary Path	(1.73 ± 0.17) · 10^4^
Blood2—> Urinary Path	(5 ± 3) · 10^–1^
Urinary Path—> Urine	(2.32 ± 0.15)

**Fig. 5:**
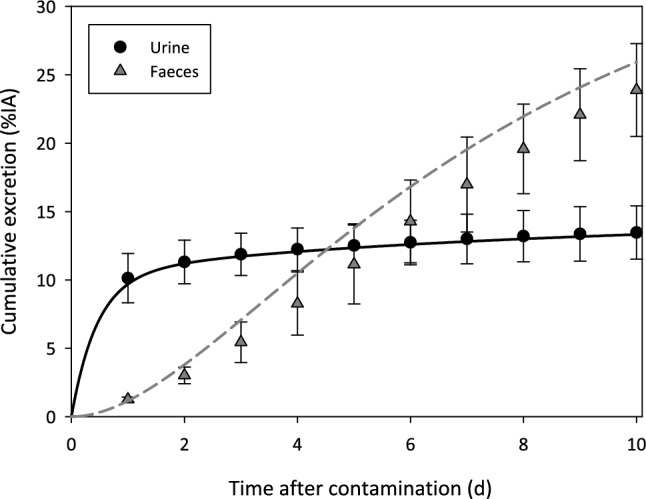
Comparison of model prediction and data for the cumulative excretion of Am in control rats. Black solid line and black dots: urine. Grey dashed line and grey triangles: faeces

Figure 5 shows the model predictions compared to the available data for Am urinary excretion (black dots and solid line) and Am faecal excretion (grey triangles and dashed line) in control rats. Figure S1 (supplementary information) shows the plasma clearance data and the corresponding model predictions.

### Assumptions for chelation

The challenging part of the CONRAD approach (Breustedt et al. 2009) is the definition of the sites where chelation occurs, which corresponds to the selection of the appropriate combinations of the interacting compartments. The proposed model for DTPA consists of the compartments “Blood”, “ECF-Slow exchange”, “ECF-Fast exchange” and “Hepatocytes” in addition to the excretion paths. The most straightforward assumption is that DTPA in the ECF compartments chelates Am in the blood compartments of the Am model and DTPA in the hepatocytes chelates Am in the liver compartments. Furthermore, the compartments that receive direct inflow from blood plasma, i.e. Liver1, Skeleton1, Kidney and ST0, are assumed to be partly associated with the ECF circulating in the corresponding tissue, so that part of the Am contained there is also available for chelation with DTPA in the ECF compartments. Therefore, all possible combinations of these compartments with DTPA in “Blood”, “ECF-Slow exchange” and “ECF-Fast exchange” were tested.

Since chelation dynamics and efficiency depend on the surrounding environment, such as the presence of other endogenous ligands or competing metals, different chelation rate constants were considered for each combination of the relevant compartments.

Based on the results of the model fits, the following combinations were found to be sufficient to describe the complete set of data:

- Am in “Kidney” is chelated with DTPA from the “Blood” compartment, but not with DTPA from the “ECF-Slow exchange” and “ECF-Fast exchange” compartments.
- Am in the “Liver1” compartment is chelated with DTPA from the “ECF-Slow exchange” and “Hepatocytes” compartments, but not with DTPA from the compartments “Blood” and “ECF-Fast exchange”.

Table 3 shows the values of the chelation rate constants as obtained in the model fits. For kR2 it was not possible to find a common value which was able to simultaneously describe the complete set of data. So the fits were performed with three different parameter values of kR2, one for each experiment (as indicated in the last column of Table 3).

**Table 3:** Values of the chelation rate constants

Chelation rate constant	Combined model compartments	Value (d^−1^ mol^−1^)	Experiment
kR1	DTPA_ECF-Slow exchange_ and Am_Liver1_	(7.2 ± 0.08) · 10^–2^	A and B and C
kR2	DTPA_Blood_ and Am_Kidney_	2.02 ± 0.06	A
		2.43 ± 0.01	B
		3.34 ± 0.11	C
kR3	DTPA _Hepatocytes_ and Am _Liver1_	16.04 ± 0.31	A and B and C

### Excretion

**Fig. 6:**
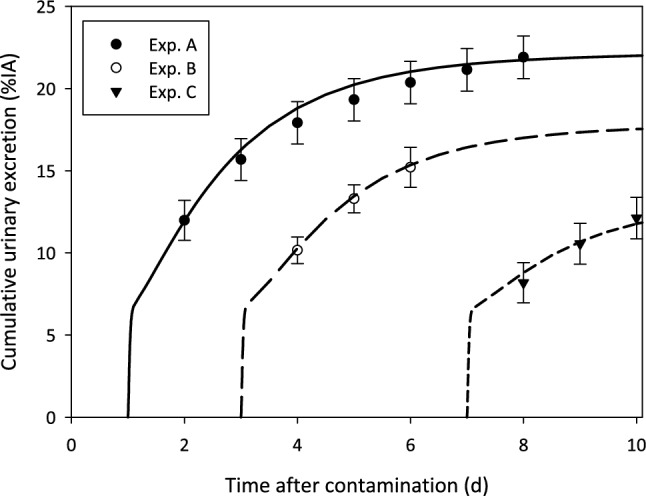
Comparison of model prediction and data for cumulative urinary excretion in chelation studies. Experiment A (300 µmol kg^−1^ DTPA on Day 1): black solid line and black dots; Experiment B (300 µmol kg^−1^ DTPA on Day 3): black dashed line and empty dots; Experiment C (300 µmol kg^−1^ DTPA on Day 7): black dashed line and black triangles

**Fig. 7:**
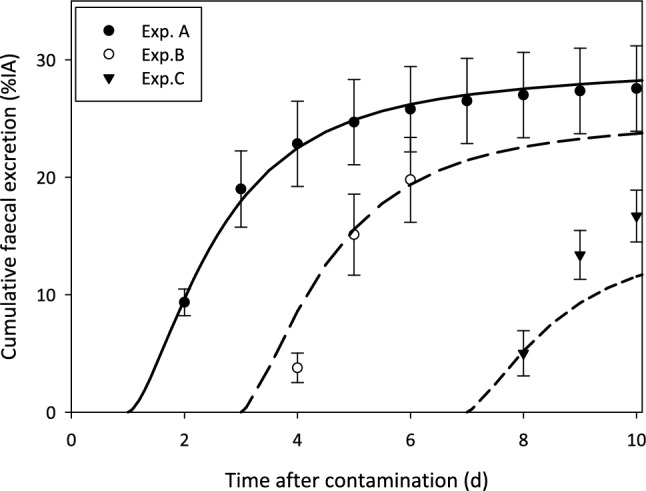
Comparison of model prediction and data for the cumulative faecal excretion in the chelation studies. Experiment A (300 µmol kg^−1^ DTPA on Day 1): black solid line and black dots; Experiment B (300 µmol kg^−1^ DTPA on Day 3): black dashed line and empty dots; Experiment C (300 µmol kg^−1^ DTPA on Day 7): black dashed line and black triangles

In Figs. 6 and 7, model predictions of the urinary and faecal excretion in the DTPA decorporation studies (Experiments A, B, C) are given, respectively. Data and model predictions are presented as cumulative excretion expressed as the percentage of the injected activity (% IA). Data were calculated as the mean values of *four* rats (mean ± SD; Table A7, Table A8).

The curves correspond to the results that fulfilled the goodness-of-fit criteria described in Sect. "Model implementation and fitting". In general, a good agreement between model prediction and data can be seen for all urine studies. The model prediction curve for faecal excretion for control rats systematically overestimates the experimental data; however, it is still within the experimental uncertainties. The curve of the faecal excretion for Experiment C cannot describe the data.

The model proved able to describe the general effect observed during the experiments, with the enhancement of urinary excretion inversely depending on the delay of DTPA administration after Am contamination.

### Organ retention

Tables 4 and 5 show the effect of DTPA treatment on Am retention in the liver and skeleton compared to untreated controls. The data are presented as the DTPA-induced reduction in Am uptake in tissues and are expressed as the mean percentage reduction relative to the corresponding untreated group (%; mean ± SD).

**Table 4:** Comparison of model prediction and experimental data on the reduction of Am uptake in rat liver after delayed DTPA treatment

	Time of measurement (d)	Time of DTPA treatment after contamination (d)	Data (%; mean ± SD)	Model (%)
Experiment A	14	1	85 ± 28	81
Experiment B	14	3	84 ± 18	84
Experiment C	14	7	82 ± 20	85

**Table 5:** Comparison of model prediction and experimental data on the reduction of Am uptake in rat skeleton after delayed DTPA treatment

	Time of measurement (d)	Time of DTPA treatment after contamination (d)	Data (%; mean ± SD)	Model (%)
Experiment A	14	1	40 ± 3	18
Experiment B	14	3	35 ± 5	15
Experiment C	14	7	24 ± 3	9

The agreement between the model and the data is in general remarkable for the liver regardless of the time of DTPA administration. On the contrary, reduction in skeleton cannot be reproduced.

## Discussion

In this study, a system of models for DTPA-induced decorporation of Am was developed based on excretion and tissue data from animal studies conducted at CEA/LRT as well as additional relevant data taken from the literature. The simplified substructures, of which this system of models is composed, describe the three available forms: the incorporated Am–citrate, the DTPA and Am–DTPA chelate.

A recent publication (Miller et al. 2019) presented a model of the systemic biokinetics of Am in rats starting from a comprehensive dataset including the pelt, liver, skeleton, lung, GI-tract, spleen, kidneys, muscle, ST and gonads. This model is based on pharmacokinetic considerations. The model parameters were assessed from the physiological knowledge of the vascular flows to the tissues and of the volumes of the extracellular fluids associated with each tissue. Extrapolation to early times was made with small uncertainties by using pharmacokinetic front-end modelling.

Figures 2 and 3 show that the data of Wedeking et al. (1990) on the kinetics of DTPA complexes, which were not used for the model fit, can also be described by the model curves. This suggests the validity of the assumption that the biokinetics of DTPA is independent of the type of chelated molecule and the ionic charge, and that the proposed structure, although simplifying much more complex processes, can successfully describe all available experimental data.

The fact that the data at our disposal originate from DTPA decorporation studies with DTPA administration at least one day after contamination proved to be the major challenge for the definition of the sites of chelation and the mechanism of the chelation process itself, since at day 1 after contamination only about 0.1% of the injected Am is present in blood. However, this enabled to focus the analysis on the long-term effects of DTPA decorporation studies. The parameter values shown in Table 3 represent the results of the model fits and the minimal set of chelation rate constants (kR) needed to describe the available data. The results are satisfactory in terms of model parsimony considering that only three chelation sites are sufficient to successfully reproduce all the studies. However, it was not possible to obtain an unique value of kR2, which is the rate constant describing the chelation of Am in the kidneys with DTPA in blood. It was possible to obtain a satisfactory fit only considering different values of kR2 for each of the three experiments, with kR2 increasing with increasing delay of DTPA tratment. This proves that the simple assumptions of second-order kinetics made in the CONRAD approach are not always sufficient to provide a globally valid set of values for the chelation rate constants. It is important to consider that the assumptions behind the model structures are simplifications of complex processes. Nonconstant kR values indicate the possibility that more complex kinetics are at stake, which, however, cannot be better modelled with available information.

According to the results, the compartment “Kidney” in the Am model is identified as one of the sites where chelation with DTPA occurs. Considering that blood makes up approximately 26.5% of the total kidney mass, according to ICRP Publication 133 (ICRP 2016), it seems reasonable to assume that the Am activity present in the “Kidney” compartment can be partly associated with blood and can thus be seen as a possible site of chelation with DTPA in blood. Similarly, the activity in the “Liver1” compartment can be associated with the ECF and therefore can be seen as a possible site of chelation with DTPA in the “Slow exchange” compartment of the ECF, as has been done before e.g. in Breustedt et al. (2019). These chelates are eliminated in the urine.

In general, it is assumed that once bound in the retention organs, the actinide is no longer physically available to the chelating agent until released by natural recycling into ECF due to loss from soft tissues. However, even though the penetration of the DTPA molecule into the cell is not an obvious assumption due to its physico-chemical properties, according to several authors, DTPA might be present in small amounts in the hepatocytes and thus be available for chelation of Am prior to bile/faeces elimination. Stevens et al. showed that as soon as 2 h after [^14^C]DTPA injection into rats, the ratio of the concentration of DTPA in the liver to that in plasma was greater than 1, reaching a maximum of 4 at 4 h, and this ratio was still greater than 3 at 48 h (Stevens et al. 1978). This indicates that DTPA molecules can penetrate liver cells, including hepatocytes, where they are retained. In addition, [^14^C]DTPA was detected in the bile of injected rats (0.12% at 24 h), thus showing the ability of DTPA to enter hepatocytes to a small extent prior to its biliary/faecal elimination (Stevens et al. 1978; Bhattacharyya 1978a; Bhattacharyya and Peterson 1979; Ballou and Hess 1972). In rats, 80–90% of the Pu present in the bile is in the form of Pu–DTPA chelate (Bhattacharyya and Peterson 1979). The only elimination pathway for the An–DTPA chelates formed inside hepatocytes is the biliary/faecal route, which is evidenced by an enhancement of faecal excretion, as shown in the data, and can be reproduced by the proposed model (see Table A8, Fig. 7). The agreement for the faecal excretion is less satisfactory than for urine. Faecal excretion data, as well skeletal retention data, were considered to be associated with substantial uncertainties and given a lower weighting in the fit process.

The available data clearly indicate the effect of delayed DTPA treatment on Pu/Am retention in bones as well. One hypothesis could be that a part of bone Am remains available for chelation: a substantial reduction in skeletal burden in a DTPA-treated human case was observed to occur in trabecular bone (James et al. 2007). Taylor (1989) showed that a Pu/Am fraction is still associated with bone surfaces for at least 7 days after rat contamination. However, tests with the proposed model involving direct chelation in the skeletal compartments were unsuccessful. Evidently, the systemic chelation assumed in the proposed model is not sufficient to reproduce the observed reduced uptake and retention in the skeleton. Additionally, in the fitting process it was not possible to find a value for the rate constants describing chelation in the skeletal compartments greater than zero. Possible reasons for this may be the large uncertainties in the available data. Activity in the skeleton was indeed estimated from measurements in two femurs by scaling the result to the whole skeleton under the assumption that the two femurs represent 10% of the total bone (Grémy et al. 2016). However, this value may show significant between subjects variations, so these results were considered less reliable than the others in the fitting process and a large variance was associated to this dataset. Additional work is needed to correctly reproduce the long-term retention of Am in the skeleton. This has only a negligible impact on the description and interpretation of the excretion data. Nevertheless, the correct reproduction of the skeletal burden would be important for the calculation of the dose, especially at later times.

## Conclusions

In the presented work, an approach for describing the unperturbed biokinetics of Am and DTPA, the chelation process and the behaviour of the Am–DTPA compound with a single model system has been applied to rat data. For this, models to describe the biokinetic behaviour of unperturbed Am and DTPA in rats were developed, which were then combined into a model system by a suitable mathematical description of the chelation mechanism as a second-order process. The proposed model system is able to describe the vast majority of excretion and tissue data from animal studies for DTPA-induced Am decorporation and for pure Am biokinetics conducted by CEA/LRT, with the exception of skeleton in all experiments. Long-term effects of chelation of DTPA following treatment at Day 1 post-contamination with Am or later can be described assuming three possible sites of chelation. For chelation in kidney the value of the chelation rate coefficient depends on the delay of DTPA treatment. Considering the scarcity of the available data and the uncertainties inherent in this kind of study, further experimental work is needed to reach a deeper understanding of the interpretation of this phenomenon.

The compartmental structure presented in this paper and developed from the CONRAD approach identified three chelation sites that allow to describe the long-term effects of DTPA treatment on the kinetics of incorporated Am with regard to excretion and reduced uptake in the liver. The analysis presented has been affected by limitations on the available data and the inability to find a valid set of common parameter values for all experiments, indicating that the simplifications inherent in the basic assumptions of the model fail to account for the complexity of the processes involved.

Nevertheless, the obtained results can contribute to improve the interpretation of biological data from DTPA-treated contamination cases and the modelling of Am decorporation by protracted DTPA treatment, and help to estimate the incorporated activity and to assess the benefit of the therapy in terms of the averted dose. Further experimental research is still needed to reach a better understanding of the physiological processes and for a future translation of the findings to human models.

### Supplementary Information

Below is the link to the electronic supplementary material.

Supplementary file1 (DOCX 38 KB)

## Data Availability

Data are available in the text and additionally as supplementary information.

## Footnotes

1 The summary of product characteristics for Ditripentat-Heyl®, including information on its authorization in Germany, can be accessed by making a search in the database of the Federal Office for Drugs and Medical Devices (Bundesinstitut für Arzneimittel und Medizinprodukte), accessible through this link: https://www.pharmnet-bund.de/dynamic/en/drug-information-system/index.html, search term: Ditripentat.
