# Supplementary material for: Modelling DTPA therapy following Am contamination in rats
Source: Radiat Environ Biophys. 2023 Oct 13;62(4):483–95. doi: 10.1007/s00411-023-01046-z (PMC10628027; doi:10.1007/s00411-023-01046-z)
Supplement: Supplementary file 1 — Supplementary file1 (DOCX 38 KB) [file 411_2023_1046_MOESM1_ESM.docx]

**Supplementary Information (SI)**

**Article title:** Modelling DTPA therapy following Am contamination in rats

**Journal name:** Radiation and Environmental Biophysics

**Author names:** Manuel Kastl, Olivier Grémy, Stephanie Lamart, Augusto Giussani, Wei Bo Li, Christoph Hoeschen

**Affiliation and e-mail address of the corresponding author Manuel Kastl:**

Institute of Radiation Medicine, Helmholtz Center Munich - German Research Center for Environmental Health

Email: manuel.kastl@helmholtz-munich.de

Phone: +49 (0) 163 3797156

Table A1 Kinetics of Am-citrate in blood of rats. Data from Turner and Taylor (1968) and from CEA/LRT studies. Data are expressed as the percentage of the administered activity (% IA; mean ± SD).

|  | n^a^ | Time after administration of Am-citrate (d) | | | | | | | |
| --- | --- | --- | --- | --- | --- | --- | --- | --- | --- |
|  |  | 6.94E-04 | 0.01 | 0.021 | 0.042 | 0.21 | 1 | 7 | 17 |
| Turner and Taylor (1968) |  | 14.9 ± 0.5 |  | 5.0 ± 0.5 | 2.2 ± 0.3 |  |  |  |  |
| CEA/LRT | 4 |  | 10.3 ± 0.5 |  |  | 0.35 ± 0.05 | 0.13 ± 0.03 | 0.03 ± 0.03 | 0.013 ± 0.05 |

^a^ n = number of rats per group; this information is not available for the study of Turner and Taylor.

Table A2 Urinary excretion of Am-citrate in control rats. Data from CEA/LRT studies are expressed as 24 hours cumulated percentage of injected activity (% IA; mean ± SD).

| n^a^ | Time after administration of Am-citrate (d) | | | | | | | | | |
| --- | --- | --- | --- | --- | --- | --- | --- | --- | --- | --- |
|  | 1 | 2 | 3 | 4 | 5 | 6 | 7 | 8 | 9 | 10 |
| 4 | 10 ± 2 | 1.2 ± 0.3 | 0.55 ± 0.07 | 0.37 ± 0.04 | 0.27 ± 0.05 | 0.23 ± 0.05 | 0.25 ± 0.22 | 0.21 ± 0.10 | 0.16 ± 0.08 | 0.11 ± 0.04 |

^a^ n = number of rats per group

Table A3 Faecal excretion of Am-citrate in control rats. Data from CEA/LRT studies are expressed as 24 hours cumulated percentage of injected activity (% IA; mean ± SD).

| n^a^ | Time after administration of Am-citrate (d) | | | | | | | | | |
| --- | --- | --- | --- | --- | --- | --- | --- | --- | --- | --- |
|  | 1 | 2 | 3 | 4 | 5 | 6 | 7 | 8 | 9 | 10 |
| 4 | 1.3 ± 0.2 | 1.8± 0.8 | 2.3 ± 0.9 | 2.9 ± 0.9 | 2.9 ± 0.8 | 3.2 ± 0.3 | 2.7 ± 0.9 | 2.6 ± 0.5 | 2.5 ± 0.7 | 1.8 ± 0.4 |

^a^ n = number of rats per group

Table A4: Pu-DTPA in blood after injection of 12.2 kBq Pu-DTPA. Data from CEA/LRT studies are expressed as the percentage of the administered activity (% IA). The uncertainty was calculated as 30 % of the measured value.

|  | n^a^ | Time after administration of Pu-DTPA (min.) | | | | | | |
| --- | --- | --- | --- | --- | --- | --- | --- | --- |
|  |  | 10 | 20 | 40 | 60 | 120 | 240 | 1440 |
| CEA/LRT | 1 | 7.14±2.14 | 4.03± 1.21 | 2.77± 0.83 | 0.91± 0.27 | 0.14± 0.04 | 0.03 ± 0.01 | 0.02 ± 0.005 |

^a^ n = number of rats per time point

Table A5: Pu-DTPA in urine after injection of 12.2 kBq Pu-DTPA. Data from CEA/LRT studies are expressed as the percentage of the administered activity (% IA). The uncertainties correspond to the measurement error, or to 3 % of the measured value (whichever the highest).

|  | n^a^ | Time after administration of Pu-DTPA (min.) | | | | | | |
| --- | --- | --- | --- | --- | --- | --- | --- | --- |
|  |  | 10 | 20 | 40 | 60 | 120 | 240 | 1440 |
| Pu-DTPA from CEA/LRT | 1 | 19.6 ± 0.6 | 20.7 ± 0.6 | 68 ± 4 | 88 ± 6 | 92 ± 8 | 91 ± 7 | 93 ± 16 |

^a^ n = number of rats per time point

Table A6: Measurements of hepatic and skeletal Am in rats on Day 14 for different DTPA treatment times. Data from CEA/LRT studies are expressed as the mean percentage of the injected activity (% IA; mean ± SD).

| Exp. | n^a^ | d  (µmol /kg) | T | Liver | Skeleton |
| --- | --- | --- | --- | --- | --- |
|  |  |  |  |  |  |
| A | 4 | 300 | 1d | 1.05±0.31 | 28±2 |
| B | 4 | 300 | 3d | 1.16±0.19 | 30±4 |
| C | 4 | 300 | 7d | 1.25±0.25 | 35±3 |

^a^ n = number of rats per group

d = dose of DTPA treatment

T = time of DTPA administration

Table A7: Urinary excretion of Am in DTPA-treated rats. Data from CEA/LRT studies are expressed as 24 hours cumulated percentage of injected activity (% IA; mean ± SD).

| Exp. | n^a^ | d  (µmol /kg) | T  (d) | Time after administration of Am-citrate (d) | | | | | | | | |
| --- | --- | --- | --- | --- | --- | --- | --- | --- | --- | --- | --- | --- |
|  |  |  |  | 2 | 3 | 4 | 5 | 6 | 7 | 8 | 9 | 10 |
| A | 4 | 300 | 1 | 12.0 ± 1.2 | 3.7±0.4 | 2.2±0.2 | 1.41±0.05 | 1.05±0.09 | 0.78±0.14 | 0.76±0.08 |  |  |
| B | 4 | 300 | 3 |  |  | 10.2 ± 0.1 | 3.1 ± 0.3 | 1.9 ± 0.9 |  |  |  |  |
| C | 4 | 300 | 7 |  |  |  |  |  |  | 8.2 ± 1.2 | 2.4 ± 0.3 | 1.6 ± 0.2 |

^a^ n = number of rats per group

d = dose of DTPA treatment

T = time of DTPA administration

Table A8: Faecal excretion of Am in DTPA-treated rats. Data from CEA/LRT studies are expressed as 24 hours cumulated percentage of injected activity (% IA; mean ± SD).

| Exp. | n^a^ | d  (µmol /kg) | T  (d) | Time after administration of Am-citrate (d) | | | | | | | | |
| --- | --- | --- | --- | --- | --- | --- | --- | --- | --- | --- | --- | --- |
|  |  |  |  | 2 | 3 | 4 | 5 | 6 | 7 | 8 | 9 | 10 |
| A | 4 | 300 | 1 | 9.4±1.1 | 9.7±3.0 | 3.9±1.6 | 1.8±0.2 | 1.11±0.13 | 0.70±0.12 | 0.51±0.10 | 0.34±0.09 | 0.21±0.01 |
| B | 4 | 300 | 3 |  |  | 3.8±1.3 | 11±3 | 4.7±1.1 |  |  |  |  |
| C | 4 | 300 | 7 |  |  |  |  |  |  | 5.0±1.9 | 8.4±0.8 | 3.3±0.8 |

^a^ n = number of rats per group

d = dose of DTPA treatment

T = time of DTPA administration

**Figure S1** comparison of model prediction (lines) and data (circles) of plasma uptake and clearance of Am in control rats; Data from Turner and Taylor (1968) plus additional unpublished data from studies conducted at CEA/LRT are expressed as the percentage of the injected activity (% IA; mean ± SD).
